# Why mothers die: Analysis of verbal autopsy data from Kersa Health and Demographic Surveillance System, Eastern Ethiopia

**DOI:** 10.7189/jogh.12.04051

**Published:** 2022-07-22

**Authors:** Merga Dheresa, Tesfaye Assebe Yadeta, Tariku Dingeta, Hirbo Shore, Yadeta Dessie, Gamachis Daraje, Abera Kenay Tura

**Affiliations:** 1 School of Nursing and Midwifery, College of Health and Medical Sciences, Haramaya University, Harar, Ethiopia; 2 Kersa Health and Demographic Surveillance Systems, Harar, Ethiopia; 3 School of Public Health, College of Health and Medical Sciences, Haramaya University, Harar, Ethiopia; 4 Department of Statistics, College of Computing and Informatics, Haramaya University, Haramaya; 5 Department of Obstetrics and Gynaecology, University Medical Centre Groningen, University of Groningen, Groningen, the Netherlands

## Abstract

**Background:**

Despite registering tremendous improvement as part of the Millennium Development Goals, Ethiopia has still one of the highest numbers of maternal mortality. Although maternal mortality is one of the commonest indicators for comparison or measuring progress, its measurement remained a challenge. In a situation where, vital registration is not in place and only few women gave birth in facilities, alternative data sources from population-based surveys are essential to describe maternal deaths. In this paper, we reported estimates of maternal mortality and causes in a predominantly rural setting in eastern Ethiopia.

**Methods:**

Data were used from the ongoing prospective open cohort of Kersa Health and Demographic Surveillance System (HDSS), located in eastern Ethiopia. At enrolment, detailed sociodemographic and household conditions were recorded for every member, followed by household visit every six months to identify any vital events: births, deaths, and migration. Whenever a death was reported, additional information about the deceased – age, sex, pregnancy status, and perceived cause of deaths – were collected through interview of the closest family member(s). Then, the probable cause of death was assigned using an automated verbal autopsy system (InterVA). In this paper, we included all deaths among women during pregnancy, childbirth or within 42 days of termination of pregnancy. To describe the trends, we calculated annual maternal mortality ratio (MMR) along with their 95% Confidence Interval (CI).

**Results:**

From 2008 to 2019, a total of 32 680 live births and 720 deaths among reproductive age women were registered. Of the 720 deaths, 158 (21.9%) were during pregnancy or within 42 days of termination of pregnancy, corresponding with an MMR of 484 per 100 000 live births. The three leading causes of deaths were pregnancy related sepsis, obstetric haemorrhage and anaemia of pregnancy. There was non-significant reduction in the MMR from 744 in 2008 to 665 in 2019, with three lowest ratios recorded in 2013 (172 per 100 000 live births), 2009 (280 per 100 000 live births) and 2016 (285 per 100 000 live births).

**Conclusions:**

There was no significant decrement of MMR during the study period. Most deaths occurred at home from pregnancy related sepsis and haemorrhage implicating the unfinished agenda of ensuring skilled delivery and appropriate postnatal management.

As part of the Millennium Development Goals, Ethiopia reduced its maternal mortality ratio (MMR) by 72% from 1990 to 2015—slightly short of the targeted 75% reduction [1]. Despite this tremendous progress, the Ethiopian MMR is still high (412 per 100 000 live births in 2016) and close to 14 000 maternal deaths occur per annum [2,3]. Although maternal mortality statistics is commonly used for comparison and/or description of progress of a country or region, effective measurement of the trends and/or ascertaining causes of deaths is impractical in several resource limited settings – where the majority of the deaths occur [4]. As such, gross national estimates and non-representative facility-based studies are common data sources for information and action [2]. However, such sources are confounded by under reporting and lack of strong registration system to capture deaths occurring outside facilities. The pathways to the global MMR target of <70 per 100 000 live births by 2030 requires strategies for capturing majority of maternal deaths and responding accordingly to minimize future preventable deaths [5,6].

As part of this, Ethiopia has introduced maternal death surveillance and response (MDSR) in 2013 to identify, review and institute responses to prevent maternal deaths [7]. However, the Ethiopian MDSR has very low coverage with only less than 10% of deaths included so far [8]. In a country where majority of births or deaths are occurring at home, facility-based systems like MDSR, with low coverage, may not give the true picture of the problem. In addition to demographic and health surveys, which periodically measures demographic and health conditions from representative population, other alternative sources like health and demographic surveillance sites (HDSS) are emerging as source of data [9]. HDSS – often affiliated with universities in Asia and Africa – are initiated to generate prospective data on demographic and health events on a continuous manner [10]. As there is a need to shift from global to local action [11], having local HDSS that would continuously generate data on demographic and health conditions of a population would have a paramount importance.

Importance of HDSS for estimating maternal mortality is well established and several new sites are being added [12]. Unlike facility-based studies which use clinician diagnosis, HDSS uses verbal autopsy for assigning of cause of deaths [13]. With the advances in time and use of verbal autopsy, automated algorithm (InterVA) for assigning cause of death is becoming common [14]. Estimation of pregnancy related mortality using of HDSS data was found to be effective in revealing the burden of mortality and its determinants across member countries in Africa and Asia [15]. In this paper, we reported maternal mortality estimates and underlying causes using verbal autopsy data from the ongoing Kersa HDSS from 2008 to 2019.

## METHODS

### Study settings

The study was conducted among reproductive age women under Kersa HDSS follow up. Kersa HDSS field site is a population-based study in a predominantly rural settings in eastern Ethiopia as described elsewhere [16]. In brief, Kersa HDSS collects basic sociodemographic and household information from all members of households in randomly selected kebeles (smallest administrative unit in Ethiopia) at enrolment. In addition, data on vital status changes—births, deaths and migration—are collected biannually. Established in 2007 by enrolling the entire population in 12 kebeles, Kersa HDSS (Kersa site) currently operates in 24 kebeles covering 25 782 households and 141 415 individuals. In the HDSS site, migration, birth, death, relationship, membership, pregnancy observation and birth outcomes are updated biannually. We included all maternal deaths that occur from January 1, 2008 to December 31, 2019.

### Study design and population

Kersa HDSS is an open dynamic cohort that longitudinally follows individuals living within the specified geographical boundary of the selected kebeles [16]. All individuals living in the HDSS are visited every six months. During each visit, all reproductive age women (15-49 years) in each household were interviewed about pregnancy and births in addition to the sociodemographic conditions collected at enrolment. In this study, we included all women who died during pregnancy, childbirth or within 42 days of termination of pregnancy and for which complete information was retrieved from the HDSS database. Maternal death was defined as per the International Classification of Disease-10 as “the death of a woman while pregnant or within 42 days of termination of pregnancy, irrespective of the duration and the site of the pregnancy, from any cause related to or aggravated by the pregnancy or its management, but not from accidental or incidental causes” [17]. Cause of maternal death was determined using verbal autopsy (VA) technique [13]. VA involves a structured interview of a closest caregiver, or relative of the deceased about details of circumstances surrounding the death to arrive at a probable cause of death. Cause of death was assigned using probabilistic approach using the InterVA approach. The InterVA algorithms assigns probable causes of deaths based on the data obtained from interview of the closest family. Unlike verbal autopsies by groups of physicians which usually assigns one more likely causes of death, InterVA generates probable causes with their respective probabilities [18-22]. As such, the cause with the highest probability will be considered the cause of death. It delivers causes of death compatible with the International *Classification of Diseases version 10* (ICD-10) [17]. Unlike the algorithm based verbal autopsy by groups of physicians – which often dichotomize causes of deaths – the interVA approach simultaneously adjust the probability of each list of causes and displays as many as three of the most probable cause, along with their associated likelihoods enabling researchers to have a have a range of differential diagnosis [21]. Based on InterVA-4, the top ten causes of death were determined.

### Data collection and measurements

Data were collected by trained HDSS data collectors through face to face interview under the supervision of trained field supervisors. Field supervisors checked completeness and quality of data on a daily basis. Once a death was confirmed, trained data collectors were redeployed for collecting detailed information regarding age of the deceased, date of death, sex, perceived cause of death, and place of death for assigning cause of death through interview of one or more of the woman’s closest relatives. In addition, additional sociodemographic conditions (residence, education status) and obstetric and reproductive health factors (age at first birth, parity, place where she gave birth, ANC visit) were retrieved from the HDSS database. Informed verbal consent was obtained from all participants.

### Data management and statistical analysis

All collected data were edited, cleaned and entered to the automated InterVA. Cause of deaths were then categorized as per the International Classification of Disease 10 (ICD-10) classification [17]. Overall and yearly MMR was calculated as maternal death per 100 000 live births along with 95% confidence interval. Cause specific mortality fraction of maternal death was presented using proportions as per the verbal autopsy classification.

### Ethical considerations

Kersa HDSS has obtained ethical clearance from the Institutional Health Research Ethical Review Committee (IHRERC) of College of Health and Medical Sciences, Haramaya University and Science and Technology Minster of the Federal Democratic Republic of Ethiopia national ethical review committee. The study was carried out in accordance with the relevant ethical guidelines and regulations when dealing with human subjects in research. Informed verbal consent was obtained from all participants.

## RESULTS

From 2008 to 2019, a total of 720 deaths in women of reproductive age and 32 680 live births were registered under the Kersa HDSS from a total 44 872 reproductive age women followed in the 24 kebeles (Kersa district). Of the 720 deaths, 158 (21.9%) were during pregnancy, childbirth or within 42 days of termination of pregnancy. The mean age of the deceased was 29(±0.7) years, ranging from 15 to 49 years. Majority of the deaths were at home (63.29%), among women with no formal education (76.58%) and housewives (72.78%) (**Table 1**).

**Table 1:** Sociodemographic characteristics of deceased women in eastern Ethiopia from 2008 to 2019 (n = 158)

Variables	Frequency	Percent
**Age at death**		
15-24	56	35.44
25-35	66	41.77
36-49	36	22.78
**Place of death**		
At home	100	63.29
In health facility	49	31.01
En route	9	5.70
**Educational status**		
No formal education	121	76.58
Read and write	4	2.53
Literate	33	20.89
**Occupational status**		
Housewife	115	72.78
Daily labourer	16	10.13
Unemployed	27	17.09
**Wealth Index (n = 149)**		
Poor	53	35.57
Middle	54	36.24
Rich	42	28.19

### Trends in Maternal Mortality Ratio

**Figure 1:**
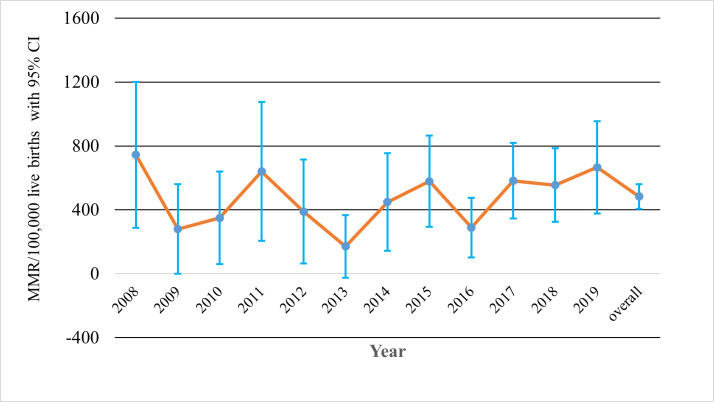
Trends in maternal mortality ratio from 2008 to 2019 in Kersa HDSS, Eastern Ethiopia. MMR – maternal mortality ratio

The overall MMR over the study period was 484 (95% CI = 411-565) per 100 000 live births. There was non-significant decrement from 744 in 2008 to 665 in 2019. The lowest MMR was recorded in 2013 (172 per 100 000 live births), 2009 (280 per 100 000 live births), and 2016 (285 per 100 000 live births) (**Figure 1**).

**Figure 2:**
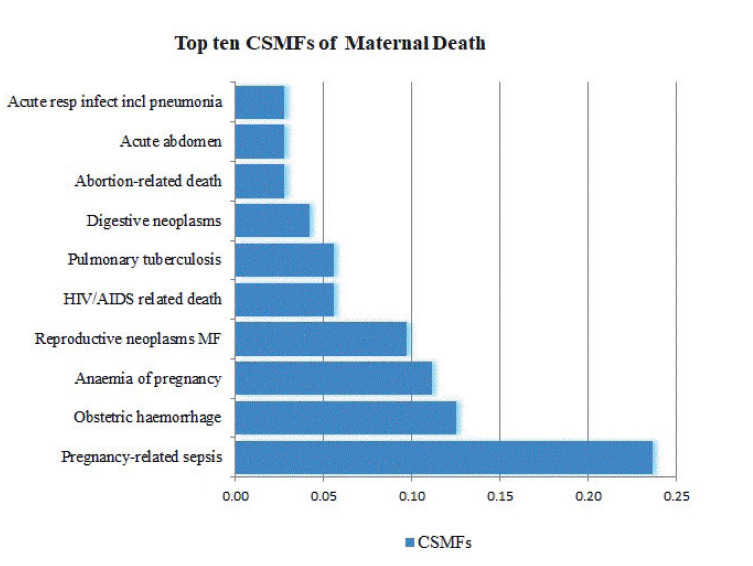
Percentage distribution of the top ten cause-specific mortality fractions as per the WHO 2012 VA cause of death categories in eastern Ethiopia (n = 158). CSMF – Cause Specific Mortality Fraction

Surprisingly, 59% of all maternal deaths were from indirect obstetric causes. The leading causes of maternal deaths as per the WHO 2012/WHO 2016 VA cause of death categories [23] were pregnancy related sepsis (24.6%), obstetric haemorrhage (12.3%) and anaemia of pregnancy (11%). The top ten cause specific mortality fraction (CSMF) are summarized in **Figure 2**.

## DISCUSSION

In this study, we reported maternal mortality in eastern Ethiopia from 2008 to 2019 using population based on the Kersa HDSS cohort. Consistent with the national figures, there was non-significant reduction in MMR during the study period. Unexpectedly, majority of maternal deaths were from indirect obstetric causes.

The estimated MMR in our study overlaps with the confidence interval of the national MMR estimate of the 2016 Demographic and Health Survey of Ethiopia [273 to 551 per 100 000 live births] [3]. The consistent stagnation in maternal mortality reduction in several low resource settings is worrisome [24-26] and requires addressing maternal mortality in an innovative way and accelerated strategies if the ambitious sustainable development goals is to be achieved by 2030 [5,6]. Moreover, ensuring strong monitoring system for maternal mortality is essential for designing appropriate and timely interventions for the last decade to 2030 [27,28].

Although Ethiopia is not immune to epidemiologic transition [29,30] and has no major shift in the obstetric transition [31], we were surprised by the fact that majority of maternal deaths in this predominantly rural setting are from indirect causes. This emergence, coupled with the absence of hypertensive disorders of pregnancy in the top ten cause of death in this cohort, implies questioning the validity of InterVA in assigning cause of deaths or the major difference in sensitivity of the algorithm between infectious disease and obstetric causes [18-20,32,33]. For several years, the leading causes of maternal deaths in Ethiopia, and other low resource settings, were from obstetric haemorrhage, hypertensive disorders of pregnancy and sepsis [8,34]. While previous studies reported high sensitivity for eclampsia (75%) and haemorrhage (75%) compared with other causes of maternal deaths [32], our finding calls for further inquiry why rare diseases like HIV/AIDS and neoplasm were higher compared with the common cause of maternal death – hypertensive disorders of pregnancy. A previous study in part of the same cohort, but covering only six years (2008-2014), which assigned cause of death by groups of physicians identified the leading cause of deaths to be postpartum haemorrhage and hypertensive disorders of pregnancy [35]. A previous study found significant difference in assigning causes of deaths between computer assisted algorithm, review teams and expert panels [36].

We found that pregnancy related sepsis was the major cause of deaths with a quarter of deaths attributed to this. Given high proportion of home deliveries in this setting, attaining hygienic delivery is unlikely and more deaths from postpartum infections is not unexpected. In addition, we found that obstetric haemorrhage is the second most common cause of maternal death. Given obstetric haemorrhage requires immediate management – stopping bleeding, fluid resuscitation, and blood transfusion – the lack of strong referral system and delays in reaching facilities from home births would contribute to the high mortality among women with haemorrhage. In addition to the ongoing work to increase institutional delivery, strengthening institutional readiness in availing blood for immediate transfusion might help in minimizing deaths from haemorrhage. Moreover, ensuring hygienic birth centres – including at home – is essential for reducing the burden of deaths occurring among home births. Since ensuring high institutional delivery is a long-term investment, improving capacity of community health workers to support families in achieving clean home births could be an essential short-term intervention. Strong community midwives – who are vigilant and could immediately refer women when required as well as skilled to assist normal births – has been found to improve maternal mortality in several countries [37,38].

Although validation of InterVA algorithm and physician diagnosis for assigning of cause of death is beyond the scope of this study, this should be further enquired. Previous validation studies reported several areas for improving the sensitivity and specificity of the algorithm [20]. Despite being praised as a remedy for maternal mortality statistics in settings with no (weak) civil registration system, HDSS requires extensive data management and linkages to identify appropriate sociodemographic and obstetric conditions associated with maternal deaths at individual level. It should also be noted that verbal autopsy diagnosis depends on several factors - respondent’s ability to observe, register and report the circumstances, symptoms and signs that lead to death, and the interviewer’s competence in probing and collecting relevant information, the algorithm’s ability to assign cause of deaths from reported illness prior to death, and associated assumptions [20]. As such, any inaccuracies in this chain of events might result in miss classification of death and should be considered during interpretation of findings from such studies.

## CONCLUSIONS

Congruent with the national figures and other similar settings, the decrement in maternal mortality in our study was non-significant. Majority of deaths occurred at home and mainly from pregnancy related sepsis, obstetric haemorrhage, and anaemia of pregnancy. Further validation study is required to understand whether the high indirect causes of deaths is attributed to the InterVA classification or other implications.
